# Anti-Stress Effects of Lemon Balm-Containing Foods

**DOI:** 10.3390/nu6114805

**Published:** 2014-10-30

**Authors:** Andrew Scholey, Amy Gibbs, Chris Neale, Naomi Perry, Anastasia Ossoukhova, Vanessa Bilog, Marni Kras, Claudia Scholz, Mathias Sass, Sybille Buchwald-Werner

**Affiliations:** 1 Centre for Human Psychopharmacology, Swinburne University, Melbourne VIC 3122, Australia; E-Mails: amygibbs57@gmail.com (A.G.); chrisneale02@gmail.com (C.N.); naomiperry21@gmail.com (N.P.); apresgrave@gmail.com (A.O.); vanessa.bilog@gmail.com (V.B.); marni.kras@monash.edu (M.K.); 2 Merck Selbstmedikation GmbH, Roesslerstrasse 96, 64293 Darmstadt, Germany; E-Mail: Claudia.Scholz@merck.de; 3 Rudolf Wild GmbH & Co. KG, Rudolf-Wild-Str. 107-115, D-69214 Eppelheim, Heidelberg, Germany; E-Mail: Matthias.Sass@wild.de; 4 Vital Solutions GmbH, Hausinger Strasse 6, D-40764 Langenfeld, Germany; E-Mail: Sybille.buchwald-werner@vitalsolutions.biz

**Keywords:** lemon balm, *Melissa officinalis*, stress, cognitive performance, functional food

## Abstract

Lemon balm (*Melissa officinalis*) has been used historically and contemporarily as a modulator of mood and cognitive function, with anxiolytic effects following administration of capsules, coated tablets and topical application. Following a pilot study with lemon balm extract administered as a water based drink, which confirmed absorption of rosmarinic acid effects on mood and cognitive function, we conducted two similar double-blind, placebo-controlled, crossover studies. These evaluated the mood and cognitive effects of a standardised *M. officinalis* preparation administered in palatable forms in a beverage and in yoghurt. In each study a cohort of healthy young adults’ self-rated aspects of mood were measured before and after a multi-tasking framework (MTF) administered one hour and three hours following one of four treatments. Both active lemon balm treatments were generally associated with improvements in mood and/or cognitive performance, though there were some behavioral “costs” at other doses and these effects depended to some degree on the delivery matrix.

## 1. Introduction

Lemon balm (*Melissa officinalis*) is a cultivated perennial lemon scented herb. Records concerning its medicinal use date back over 2000 years, including a recommendation by Paracelsus (1493–1541) that lemon balm would completely revivify a man and should be used for “all complaints supposed to proceed from a disordered state of the nervous system”. Several herbal apothecaries have attributed the plant with general beneficial effects upon the brain including specific improvements to memory [1].

More recently research attention has turned to the bioactive properties of *M. officinalis*, including its central nervous system effects. Regarding its neurocognitive effects, cholinergic nicotinic and muscarinic receptor binding in human brain homogenates varied considerably across strains of *M. officinalis* [2]. An extract with negligible cholinergic receptor binding produced, in humans, behavioural results consistent with its long traditional use as a mild sedative/anxiolytic but did not enhance memory [3]. Conversely an extract screened for high muscarinic and nicotinic binding in human brain tissue had the same calming effects but also improved memory performance [4]. This suggests that, in the case of *M. officinalis,* the robust calming/anxiolytic effects of the plant [3,4,5] may be dependent on an, as yet unidentified, non-cholinergic mechanism. An overview of these studies can be found in the review by Kennedy and Scholey [1].

In the case of cholinergic receptor binding alone, whilst muscarinic and nicotinic binding have been demonstrated, it is unclear whether this occurs at receptor sub-types with known effects on cognition, such as the muscarinic M1, M2 and M4 receptors, and nicotinic α4β2 and α7 receptors. The mood/anxiolytic effects of lemon balm may be attributable to known interactions with GABA-A receptors [6].

To date studies into the psychogenic effects of *M. officinalis* have been limited to the use of extracts administered in capsules [4], coated pills [5] or applied topically [7]. One obvious next step in such studies is to evaluate the effects of bioactive nutrients delivered in real foodstuffs as these more palatable delivery systems may increase compliance if *M. officinalis* were to be used in a health context. Here we report two studies into the anti-stress and cognitive effects of *M. officinalis* delivered in a beverage and in a yoghurt drink. The beverage was an ice-tea link water based beverage with similar bioavailability to a capsule formulation, as the extract is water soluble. The second study used a more complex food matrix, an emulsion, in a drinkable yoghurt.

In order to formulate a product which could be compared with placebo, without compromising nutritional value, a natural fruit sweetener was used. Thus as well as lemon balm, the products contained the natural fruit sweetener, Fruit Up^®^. Its complex carbohydrate composition provides a low glycaemic index of 39 ± 7. Fruit Up^®^ has no flavour itself but is characterized by a natural sweet sensation in the final product. The main carbohydrates in Fruit Up^®^ are glucose, fructose, sucrose and naturally occurring polyols. It is also known that carbohydrates and glucose can improve cognitive function. In the case of glucose this may be due to the provision of additional metabolic resources to central processes underpinning cognitive performance [8,9]. It has also been shown that lower glycaemic index foods can improve cognitive function [10], thus a low GI food containing lemon balm may afford further benefits to cognition.

Mild, measurable stress can be induced in humans in the laboratory in a variety of ways. These include via participants performing “multi-tasking” activities where mental resources are taxed while attempting to meet ongoing cognitive goals. The Purple Multi-tasking Framework (MTF), previously known as the Defined Intensity Stress Simulator (DISS), has been developed as a platform for eliciting acute psychological stress. Previous research has shown that performance of the MTF reliably increases self-ratings of negative mood and anxiety, and results in stress-related physiological responses [5,11,12,13,14,15,16]. The MTF has specific advantages over other laboratory stressors such as the Trier Social Stress Test (involving simulated public speaking). Firstly it can be repeated on a number of occasions, allowing its use in cross-over design experiments such as the two reported here. Secondly it produces a number of outcomes which allow a concomitant assessment of cognitive outcomes, in this case psychomotor, memory and attentional performance. 

The primary aim of the present two studies was to evaluate the mood and cognitive effects of two lemon balm-containing products (a tea-like beverage in Study 1 and a yoghurt drink in Study 2). We hypothesised that, compared with control treatment, the lemon balm containing treatments would reduce negative mood responses to multi-tasking. These included the negative mood/anxiety and the physiological stress response (measured as salivary cortisol) induced by the multi-tasking battery. Any effects on cognitive performance during completion of the battery were also examined. The studies were also designed to delineate any contributing effects of other potentially psychogenic constituents contained within the foods.

## 2. Method

Following a pilot study, two behavioural studies followed a randomised, placebo-controlled, balanced crossover design aimed at examining the mood, cognitive effects and anti-stress effects of lemon balm preparations. The trial was registered on the Australian New Zealand Clinical Trials Registry as ACTRN12609000864224.

### 2.1. Pilot Study

A single-blind pilot study was conducted to assess the effect of lemon balm on cognition in subjects with self defined stress and to examine the bioavailability of potential bioactive substrates of lemon balm.

#### 2.1.1. Participants

Five male or female subjects aged 23–28 were recruited for the study on the basis of considering themselves to lead a stressed lifestyle.

#### 2.1.2. Treatments

The lemon balm extract was obtained by extraction using cut dried leaves with water at 60 °C under stirring with subsequent filtration. The filtrate was then evaporated under vacuum at 40 °C. The resultant native extract was standardized with Maltodextrin to a rosmarinic acid content of >2%. An iced-tea type drink containing a standardised, extract of *M. officinalis* was supplied by Wild (Wild GmbH & Co., Heidelberg, Germany). On the testing day, participants received an identical 200 mL serving containing 1.8 g lemon balm extract standardized to 2% rosmarinic acid and Fruit Up^®^ natural fruit sweetener.

#### 2.1.3. Procedure

Participants visited the laboratory on two occasions. The first was a training day which was similar to study day, with the exception that no treatment was given. The second was the testing day, on which participants refrained from consuming any food for 10 h prior to consuming the test sample. Cognitive and mood modulating effects were evaluated using the Cognitive Drug Research (CDR) core battery, a computerised cognitive assessment system. The battery covers the cognitive domains of attention/concentration, short term working memory, long term secondary memory. Mood was also assessed. The battery takes 20–25 min to complete and consists of tasks testing several cognitive domains (see [17] for details). These include attention (simple reaction time, choice reaction time, digit vigilance) working memory (numeric working memory, spatial working memory) and secondary memory (word recall, word recognition and picture recognition). Mood was assessed using a computerized version of the Bond-Lader Visual Analogue Scales (VAS) questionnaire [18] which records aspects of mood. In addition two traditional, validated pencil-and-paper questionnaires were used to assess mood, the Profile of Mood States (POMS) and the Spielberger State Anxiety Questionnaire [19,20]. Blood samples were taken at baseline, 30 minutes, and 2, 3, 4, 6, 8 and 12 hours post consumption of the test food. Rosmarinic acid was chosen as a biomarker for bioavailability. The pilot study was conducted by RSSL, The Science and Technology Centre, The University of Reading, UK who acted as an independent Contract Research Organisation (CRO) to produce the pilot data.

### 2.2. Study 1. Effects of Lemon Balm in Drinks on Stress and Performance

#### 2.2.1. Participants

Twenty-five participants (17 female and 8 male) were recruited via advertisements in local newspapers and university bulletin boards. Ages ranged from 18 to 39 years (M = 25.3, SD = 6.2). All participants reported that they were in good health, were not taking any drugs or medications (excluding the contraceptive pill), had no known food allergies or allergies to natural sweeteners, and were non-smokers.

The study had approval from the Swinburne University Human Research Ethics Committee and all participants gave written informed consent. The study was conducted in accordance with the Declaration of Helsinki. Participants received a payment of 200 AUD at the end of the study for their participation.

#### 2.2.2. Treatments

The lemon balm extract was obtained by extraction using cut dried leaves with water at 60 °C under stirring with subsequent filtration. The filtrate was then evaporated under vacuum at 40 °C. The obtained extract had a rosmarinic acid content of >6%. This increased level of rosmarinic acid allowed the development of products containing higher concentrations for studies 1 and 2.

An iced-tea type drink containing a standardised, extract of *M. officinalis* (produced by Cognis for this study and commercially available as Bluenesse^®^ from Vital solutions) was prepared and supplied by Wild (Wild GmbH & Co., Heidelberg, Germany). On each testing day, participants received an identical 480 mL serving containing either (1) 0.3 g lemon balm and natural fruit sweetener, (2) 0.6 g lemon balm and natural fruit sweetener; or (3) 0.6 g lemon balm and a blend of artificial sweeteners (containing cyclamate, asparatame, acesulfame-K and saccharine) or (4) a placebo beverage containing the same blend of artificial sweeteners, flavoured and coloured to match the appearance of the other drinks. Treatment blinding was performed by a disinterested third party who played no further part in the study.

#### 2.2.3. Procedure

Participants visited the laboratory on five occasions. The first was a practice day during which the procedure was similar to the other four study days, with the exception that no treatment was given and participants only performed the tasks once. These data were not analysed other than to ensure that each participant’s performance lay within an acceptable range of scores for this population. Before the first study day, a disinterested third party, using random number tables, allocated participants to a treatment regimen dictated by a Latin square, which counterbalanced the order of treatments across the 4 testing days of the study. Each of the 4 testing days was separated by a 7-day “washout” period. All testing took place in small groups (3–6 participants) in dedicated laboratory facilities at the same time on each day.

On all of the four testing days participants refrained from drinking anything other than decaffeinated products on the morning of the day of the study. On the testing days, participants refrained from consuming caffeinated products and were required to eat a light breakfast, the nature of which was recorded and participants were requested to eat a similar meal on subsequent visits. They were also provided with the same type of sandwich for lunch (with either chicken and salad, or cheese and salad). On testing days, sessions began at the same time for each volunteer. In order to control for diurnal changes in cortisol these commenced at the same time for each participant in the afternoon or evening sessions (either 12 pm or 4 pm respectively). During each testing day, participants completed three “blocks” of testing. Each “block” consisted of STAI-S, saliva sample, word presentation, immediate word recall, Bond-Lader, stress and fatigue VAMS, 20-min MTF, Bond-Lader, stress and mental fatigue VAMS, delayed word recall, word recognition, saliva sample, and STAI-S.

At the beginning of the testing day, participants completed the STAI-T and the DASS immediately followed by the first “block” to establish baseline performance and stress reactivity on that day. Participants were then given the day’s treatment (active or placebo) and instructed to eat their lunch. The other two “blocks” were completed 1-h and 3-h post treatment. At the end of the last “block”, participants additionally completed the symptom checklist.

#### 2.2.4. Cognitive and Mood Measures

##### 2.2.4.1. Multi-Tasking Framework

Participants were required to simultaneously perform four tasks presented via computer. The multitasking interface (Purple Research Solutions Ltd., UK) was presented on Dell computers with high definition screens. Participants were instructed via on screen standard instructions to attend simultaneously to all four tasks, while monitoring the central counter displaying their accumulated aggregate score. Scores were dictated by speed and accuracy, with failure to respond resulting in negative scoring. Previous research has shown that simultaneous performance of these four tasks engenders increases in subjective stress and frustration and stress-related physiological responses, including an increase in salivary cortisol [14]. In the current study, all four tasks were set at a medium difficulty/intensity level and were performed for 20 min.

**Figure 1:**
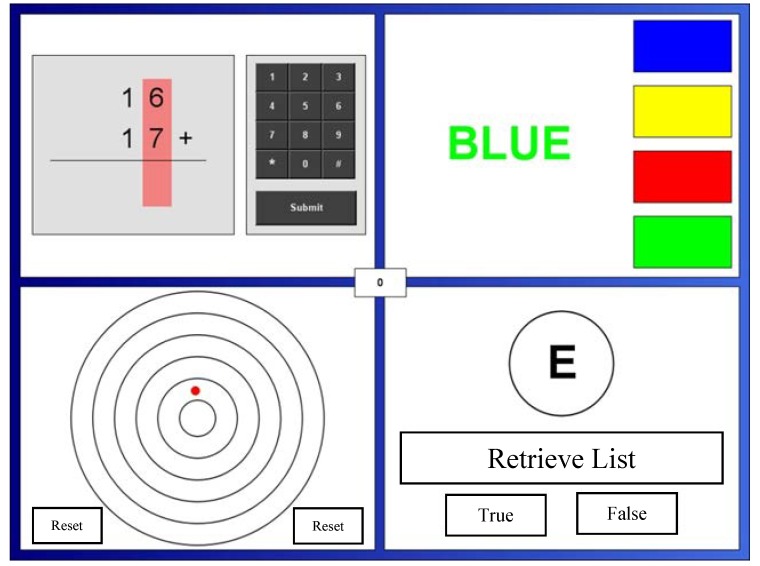
The on-screen layout of the Multi-tasking framework. In this case depicting (clockwise from top left) mental arithmetic, stroop, memory search, and psychomotor tracking.

The configuration of the tasks is shown in Figure 1 and described below.

##### 2.2.4.2. Mental Arithmetic

A series of arithmetic problems (addition of two three-digit numbers) are presented (note Figure 1 shows a two-digit version ). Using a number pad to the right of the sum, participants use the mouse to enter the answer. They are instructed that in the case of any error they should click on the digit that they wished to change and then use the number pad to select a new answer. When the volunteer is satisfied with the answer they click “Done” whereupon a new sum appears. Ten points are awarded for each correct answer and 10 points subtracted for an incorrect answer.

##### 2.2.4.3. Stroop

The Stroop task [21] is a classic psychological test of selective attention and response inhibition. In the current form, four colour blocks (Blue, Yellow, Red and Green) appear on the right hand side of the task (see Figure 1). At a given time interval, a colour name appears to the left of the colour blocks. The task is to click the colour block on the right related to the font colour, regardless of the colour it describes (e.g., the correct response would be the green block to the colour name “blue” appearing in a green font). Ten points are added for every colour word correctly identified, and 10 points subtracted for each incorrect answer, or for not making a response in the allotted time period (20 s).

##### 2.2.4.4. Memory Search

This task is adapted from the original Sternberg working memory task [22]. It measures performance of working memory (where information is held in consciousness). An array of four letters appears for the participants to remember. After 4 s, the letters disappear (but can be viewed again by clicking on “retrieve list” button). Approximately every 10 s, a single target letter appears. Participants indicate whether the target letter had appeared in the original list of four letters by clicking on the “yes” or “no” buttons. Ten points are awarded for a correct answer, 10 points deducted for an incorrect answer or no response. Five points are deducted every time the list is retrieved.

##### 2.2.4.5. Psychomotor Tracking

This task assesses psychomotor ability. A small dot drifts outwards at a speed of five pixels per second from the centre of a target comprising five concentric circles (Figure 1). The participant is instructed to allow the dot to travel as far out of the centre as possible, without letting it hit the edge of the target, before clicking on the “reset” button. Two points are added to the running total for every line that the dot passes (with a maximum of 10 points), with a penalty of 10 points for every half second that passed between the dot hitting the outer edge and the participant clicking on the “reset” button.

#### 2.2.5. Mood Scales and Other Pencil- and -Paper Measures

##### 2.2.5.1. State-Trait Anxiety Inventory

The State-Trait Anxiety Inventory (STAI) [20] consists of two scales. The “Trait” (STAI-T) subscale comprises 20 different statements (e.g., “Some unimportant thought runs through my mind and bothers me”). Participants are asked to indicate how they generally feel on a scale ranging from “almost never” to “almost always”. The “State” (STAI-S) subscale is a widely used instrument for measuring fluctuating levels of anxiety. The subscale contains 20 statements (e.g., “I am calm”). Participants rate how much they feel like each statement at the time of making the response by marking a 4-point scale ranging from “not at all” to “very much so”. Scores on both sections of the STAI range from 20 to 80, with higher scores indicating more anxiety. 

##### 2.2.5.2. Bond-Lader Visual Analogue Scales

A computerised version of The Bond-Lader mood scale [18] consisted of 16 single-screen visual analogue scales with the end points anchored by antonyms: alert-drowsy, calm-excited, strong-feeble, muzzy-clearheaded, well coordinated-clumsy, lethargic-energetic, contented-discontented, troubled-tranquil, mentally slow-quick witted, tense relaxed, attentive-dreamy, incompetent-proficient, happy-sad, antagonistic-friendly, interested-bored, withdrawn-sociable. Using a computer mouse, participants were required to place a cross along the line of the visual analogue scale indicating how they felt right now. Once satisfied with their decision, participants were required to click “next” to move to the next scale. Scores are calculated as recommended by the authors to form three mood factors: “alert”, “calm” and “content”.

An additional single scale required the participant to indicate their current levels of stress on a line with the end points labelled “not at all” and “extremely”. Scores were calculated out of 100 with higher scores reflecting higher subjective feelings of stress.

##### 2.2.5.3. Depression Anxiety and Stress Scale (DASS)

The shortened 21-item version of the DASS [23] was used to assess three negative affective states of depression, anxiety and stress on seven-item scales. The Depression subscale (DASS-D) measures symptoms relating to dysphoric mood (e.g., sadness), for example “I couldn’t seem to experience any positive feeling at all”. The Anxiety subscale (DASS-A) assesses symptoms associated with physiological hyperarousal such as autonomic arousal, for example “I felt I was close to panic”. The Stress subscale (DASS-S) assesses symptoms associated with nervous arousal, for example “I tended to over-react to situations”. Participants were required to indicate on a 4-point scale whether each statement applied to them not at all, to some degree, a considerable degree, or most of the time. Scores were calculated by summing the scores of the appropriate items. Good internal consistency and validity for the DASS have been found with samples of clinical patients and non-clinical volunteers [24].

##### 2.2.5.4. Symptom Checklist

The symptom checklist consisted of 28 physiological/psychological problems people might have (e.g., I feel dizzy, I have a dry mouth, I feel anxious more than usual). Participants indicated how much the problem had bothered them in the last 7 days including today using a 5-point scale from not at all to very much so.

#### 2.2.6. Cortisol Measurement

The participants provided salivary samples using salivettes (Sarstedt, Leicester, UK) as a non-invasive measure of free, bio-available cortisol levels [13]. Participants were required to place a cotton dental roll in their mouth and chew on it for approximately 30 s, these were immediately frozen at −20 °C. Samples were defrosted and centrifuged prior to testing for cortisol levels by luminescence immunoassay according to the instructions of the manufacturers (IBL Hamburg, Flughafenstrasse 52a, D-22335 Hamburg, Germany).

#### 2.2.7. Memory Measures

##### Word Recall/Recognition

Tests of recall/recognition measured secondary/declarative memory (where information is encoded and then retrieved). Ten words were presented centrally on the computer screen for two seconds with an inter-stimulus interval of one second. Immediately after, participants were instructed via on screen instructions to write down as many words as they could remember from the list that was presented to them (immediate condition). Approximately 20–25 min later, participants were instructed via on screen instruction to again write down as many words as they could remember from the list that was presented to them (delayed condition). For the word recall, 20 words (10 from the original list and 10 novel words) were presented centrally on the computer screen. Participants were required to use the mouse to indicate “yes” the word was in the original list or “no” the word was not in the original list. Accuracy and response time measures were recorded.

### 2.3. Study 2: Effects of Lemon Balm Yoghurt on Stress and Performance

#### 2.3.1. Participants

Twenty-one participants (8 female and 13 male) were recruited via advertisements in local newspapers and university bulletin boards. Ages ranged from 21 to 30 years (M = 23.6, SD = 3.0). All participants reported that they were in good health, were not taking any drugs or medications (excluding the contraceptive pill), had no known food allergies or allergies to natural sweeteners, and were non-smokers.

The study had approval from the Swinburne University Human Research Ethics Committee and all participants gave written informed consent. The study was conducted in accordance with the Declaration of Helsinki. Participants received a payment of 200 AUD at the end of the study for their participation

#### 2.3.2. Treatments

The lemon balm was extracted as described in section 2.2.3. Yoghurts containing the standardised extract of *M. officinalis* were supplied by Wild (Wild GmbH & Co., Heidelberg, Germany). On each testing day, participants received an identical 250 g yoghurt containing either (1) 0.3 g lemon balm extract sweetened with a natural fruit sweetener as described in Study 1; (2) 0.6 g lemon balm extract with a natural fruit sweetener; (3) 0.6 g lemon balm extract with the blend of artificial sweeteners described in Study 1 or (4) a yoghurt without lemon balm with the artificial sweetener blend. All treatments were matched for colour, flavour and appearance. The treatments were blinded by a disinterested third party who played no further part in the study.

#### 2.3.3. Procedure

All testing and outcome measures were identical to those described in sections 2.2 with the exception that the Bond-Lader mood scales used in Study 2 were a pencil-and-paper version made up of 16 × 100 mm visual analogue scales presented on a single A4 sheet of paper. The same antonyms and scoring method was used as in Study 1. The Stress visual analogue scale used in Study 2 was a pencil-and-paper version and included an additional Fatigue visual analogue scale (“How mentally fatigued do you feel right now?”). The scales consisted of two 100 mm lines with end points labelled “Not at all” and “Extremely” with each question written above one line. Participants were instructed to mark on the scale how they felt at that moment in time.

### 2.4. Statistical Treatment

To ensure no bias due to baseline performance differences a series of one-way ANOVAs were performed on each outcome comparing baseline performance prior to each of the treatments. There were no significant baseline differences in any measure of mood or performance.

Outliers were defined as any individual score which lay more than three standard deviations from the mean. These were removed prior to analysis (Ns analysed can be inferred from degrees of freedom). The data were analysed using the same approach as previous studies using this methodology. For each performance measure change-from-baseline scores at 1 h and 3 h were analysed. For each mood measure and salivary cortisol, the change during baseline MTF was subtracted from the change at 1 h and at 3 h to derive a “change-from-baseline-change” figure for each time point. The resulting scores were subjected to a two-way (Treatment × Time) repeated measures ANOVA with 4 levels of the former and two levels of the latter. Any significant main effects of Treatment or Treatment × Time interactions were explored, as were strong trends from the initial ANOVA (*i.e.*, *p* < 0.06) using paired *t*-tests as described previously [3,4,5,11,12]. To protect against Type I error, further analyses were restricted to pre-planned comparisons of each condition with the corresponding placebo at each time point.

## 3. Results

### 3.1. Pilot Study

**Figure 2:**
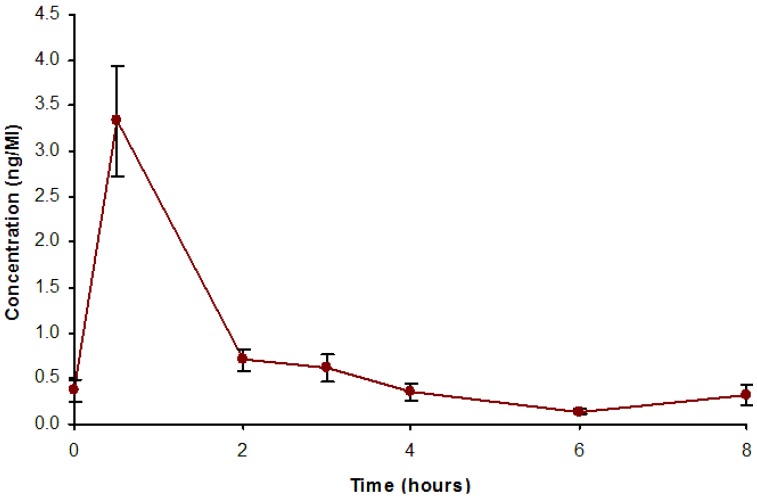
Serum concentration of rosmarinic acid following oral administration of lemon balm (means ± SEM are presented).

The bioavailability data from the pilot study are presented in Figure 2. From this figure it can be seen that rosmarinic acid from lemon balm is bioavailable, peaking within 1 h before returning to baseline levels relatively rapidly.

The pilot study also involved collection of cognitive and mood data at 0.5, 2, 3, 4, 6, 8 and 12 h post administration. These indicated possible improvements in word recognition at 2 and 6 h, alertness and 3 and 4 h, contentment at 8 and 12 h and decreased anger at all time points.

### 3.2. Study 1

**Figure 3:**
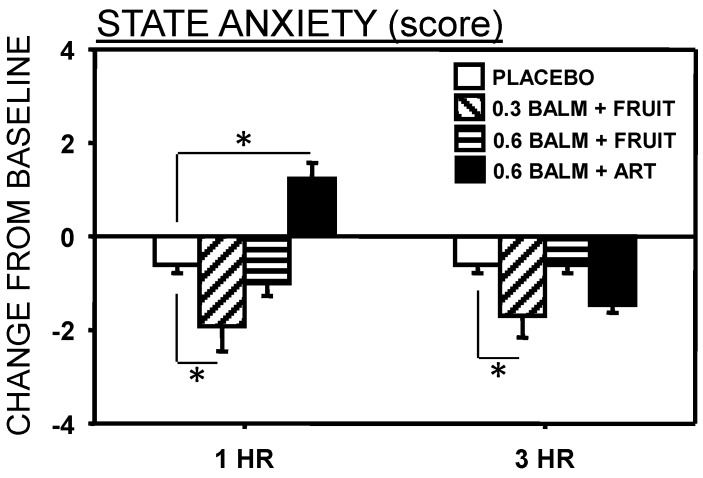
Effects of lemon balm on state anxiety (means ± SEM are presented). Significant differences between treatments are indicated by asterisks (* *p* < 0.05).

For State anxiety there was a significant Time x Treatment interaction (*F*(3,30) = 3.55, *p* = 0.026). There was a significant reduction in state anxiety following the 0.3 g lemon balm/fruit sweetener drink at both 1 and 3 h post drink (Figure 3). Conversely at 1 h the 0.6 g lemon balm/artificial sweetener was associated with greater state anxiety. No other mood effects were significant.

**Figure 4:**
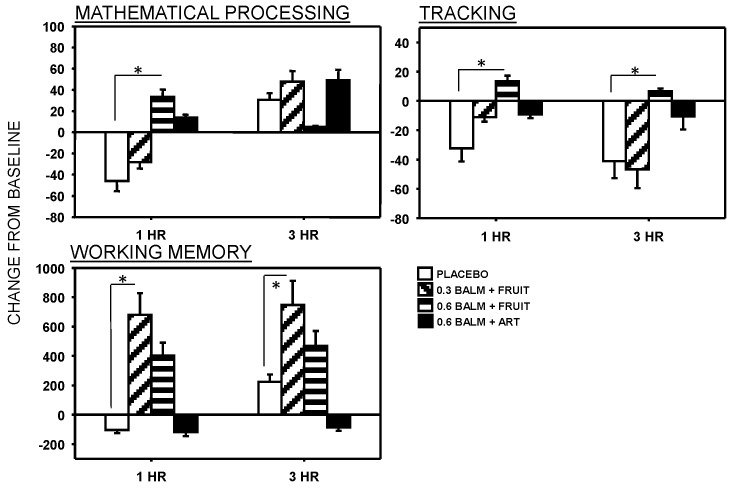
Effects of lemon balm drinks on performance of the multi-tasking framework (means ± SEM are presented).

**Figure 5:**
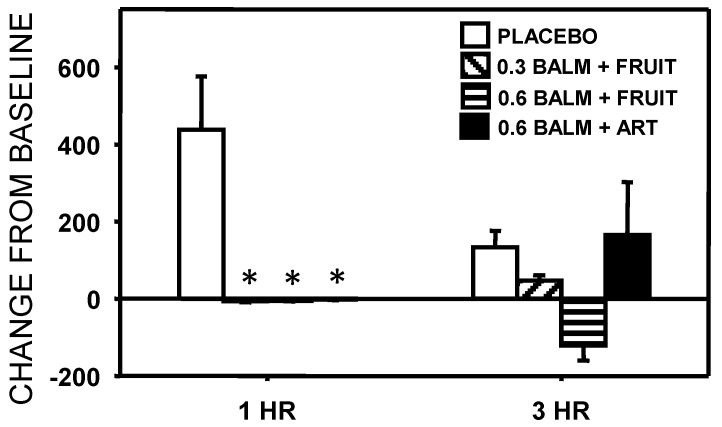
Effects of lemon balm drinks on salivary cortisol levels (means ± SEM are presented).

There were a number of significant effects of for the cognitive outcomes (Figure 4 and Figure 5). For Mathematical processing there was a significant Time x Treatment interaction (*F*(3,48) = 4.058, *p* = 0.012). Compared with placebo, this measure was significantly improved by the 0.6g lemon balm/fruit sweetener drink at 1 h only. For tracking, there was a significant main effects of treatment (*F*(3,30) = 5.300, *p* = 0.005) with performance being significantly improved over placebo by the 0.6 g lemon balm/fruit sweetener drink at both 1 h and 3 h. For the working memory measure, there was a trend towards a significant main effect of treatment (*F*(3,42) = 2.773, *p* = 0.056) with performance being significantly improved over placebo by the 0.3 g lemon balm/fruit sweetener drink at both 1 h and 3 h.

There was a significant Time × Treatment interaction for salivary cortisol levels (*F*(3,27) = 3.053, *p* = 0.045). Cortisol was elevated 1 h post-treatment in the placebo condition, this elevation was not apparent in any active treatment condition (the differences were statistically significant).

### 3.3. Study 2

**Figure 6:**
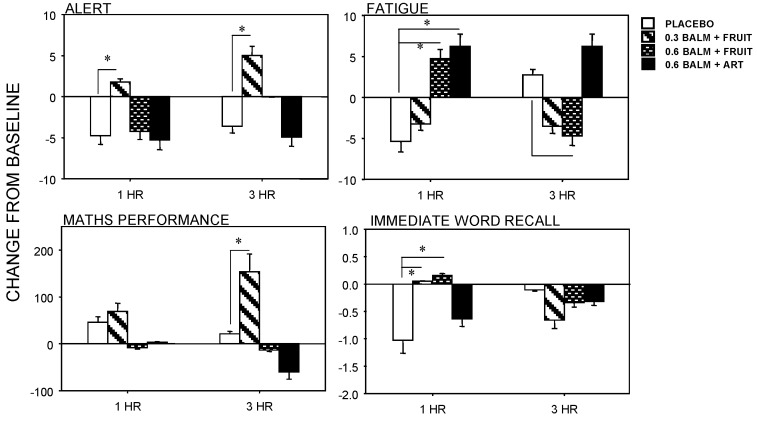
Effects of lemon balm in yoghurt on alertness, mental fatigue, maths performance and immediate word recall (means ± SEM are presented).

The significant findings from Study 2 are presented in Figure 6. For alertness there was a trend for a Treatment effect (*F*(3,54) = 2.732, *p* = 0.053), with performance being significantly better following 0.3 g lemon balm with fruit sweetener at both 1 h and 3 h. For mental fatigue there was a significant Treatment × Time interaction (*F*(3,48) = 4.142, *p* = 0.011), with significantly greater fatigue following both 0.6 g lemon balm with fruit sweetener and artificial sweetener at both 1 h, and significantly reduced fatigue for the 0.6 g lemon balm with fruit sweetener at 3 h only. Regarding maths performance, there was a significant main effect of Treatment (*F*(3,45) = 5.629, *p* = 0.022) and a significant Treatment × Time interaction (*F*(3,45) = 2.924, *p* = 0.044). Compared with placebo, maths performance was significantly improved by the 0.3 g lemon balm preparation at 3 h only. There was a significant Treatment × Time interaction for Immediate Word Recall (*F*(3,51) = 2.835, *p* = 0.047), with both the 0.3 and 0.6 g lemon balm with fruit sweetener treatments being associated with better word recall at 1 h only.

## 4. Discussion

These results demonstrate that the lemon balm products were capable of benefiting a number of aspects of mood and performance. Moreover in the case of the lemon balm drinks this appears to be the case with minimal behavioural “costs”.

Regarding the drink preparations used in Study 1, compared with placebo, the drink containing 0.3 g lemon balm and fruit sweetener was associated with lower state anxiety and better working memory at both 1 and 3 h post-ingestion. This is a very promising behavioural profile for a number of reasons. At this dose it appears that the lemon balm drink is capable of anxiolysis for a sustained period. Such an effect is similar to that observed with more mainstream anxiolytics such as benzodiazepines. However in the case of pharmaceutical anxiolytics one might also expect impairments to performance, including reductions in psychomotor and working memory capabilities [25]. In the case of the 0.3 g lemon balm drink, there was no impairment to psychomotor performance (as assessed by tracking scores) and working memory performance was significantly improved by the same treatment at both 1 h and 3 h. This profile is somewhat different to previous work using lemon balm in tablet or capsule form. Using a similar paradigm to the current study, we found increased calmness following one dose of lemon balm and while there were no cognitive costs associated with that dose, there were also no cognitive benefits—although a different dose improved mathematical processing scores [5].

As in the previous study [5], here a different, higher (0.6 g) dose of lemon balm also improved mathematical processing (at 1 h only). The same dose also significantly improved psychomotor performance, as assessed by the tracking module, at both post-drink time points. The different profile of behavioural effects suggests that at higher doses different mechanisms might be involved.

For Study 1 there was a single treatment-associated negative effect. The 0.6 g lemon balm with artificial sweetener resulted in higher state anxiety at 3 h post-drink. Since this effect was not evident in the equivalent lemon balm dose with natural fruit sweetener we can probably attribute this effect to the presence of artificial sweetener, an effect which has been observed with longer term artificial sweetener use [26], or to some interaction between the sweetener and lemon balm.

These changes were complemented to some degree by the cortisol responses. Compared with placebo, all three active treatments were associated with reduced cortisol at 1 h. Though not statistically significant, this numerical difference was still evident for the 0.3 g dose at 3 h, whereas the 0.6 g dose was reduced below baseline. The differential effects on cortisol levels suggest that, at higher doses different mechanisms might be involved—although we do not wish to over-interpret these data. It would be of interest to examine the effects of even higher doses of lemon balm to determine whether they reduce cortisol further.

The effects in Study 2 were less straightforward, with the benefits from lemon balm yoghurt preparations associated with some costs. In Study 2, there was an indication of benefits from the lower (0.3 g) dose improving alertness and word recall at 1 h with the alertness benefit remaining and maths performance also being improved at 3 h. On the other hand both the higher 0.6 g lemon balm yoghurts were associated with more fatigue at 1 h with this effect being evident at 3 h for the 0.6 g lemon balm with natural fruit sweetener preparation. 

Thus, while the 0.3 g lemon balm + fruit sweetener yoghurt may improve both alertness and memory functioning, other doses may have negative behavioural effects. It is also worth noting that the memory effects of the two preparations differed. In the case of the fruit drink working memory (where information is held “online” in consciousness) was improved by 0.3 g lemon balm at both 1 h and 3 h. In the case of the yoghurt preparation, at 1 h both 0.3 g and 0.6 g lemon balm with fruit sweetener were associated with better word recall—an aspect of declarative memory involving encoding and retrieval of information. In the latter case it may be useful to investigate which of these aspects of memory (encoding, recall,* etc.*) are most affected by the lemon balm. It should also be noted that the solubility of lemon balm in yoghurt is more complex than in a water-based drink. Yoghurts are emulsions consisting of water and fat phases. The investigated lemon balm extract, obtained by water extraction, is soluble in water and has limited solubility in fat. This may also affect the bioavailability of the bioactive compounds in lemon balm extract, consequently leading to different effects.

The mechanisms by which lemon balm imparts the above effects are unknown. For example the striking reduction in subjective ratings of state anxiety with improved aspects of performance is not easily explained by single mechanisms (e.g., individual neurotransmitter systems). Previous investigations have demonstrated variable nicotinic muscarinic binding properties for a range of lemon balm preparations [7]. Thus while cholinergic modulation may play a role in the effects seen here (in particular the enhancement of memory and alertness), direct measurement of this would be required to make any definitive conclusion. Given the anxiolytic properties of the lemon balm extract, the GABA-ergic system is one candidate target for its effects. Certainly recent research in this area points to GABA-ergic modulation by lemon balm—possibly via inhibition of the enzyme GABA transaminase (GABA-T) [6]. GABA-T is a common target for anxiolytic drugs and it has recently been found that rosmarinic acid from lemon balm has potent anti-GABA-T activity. Again direct receptor binding assays would be necessary to draw any firm conclusions. However this mechanism is supported by the pilot study showing that rosmarinic acid, one of the actives within the preparation used in the current study, peaks at around 30 min post-ingestion. This is consistent with positive effects on a number of parameters measured here including reduced cortisol in Study 1. However the cortisol data should be treated with some caution as there were a large number of missing data points for this measure (nevertheless the effects were significant).

Furthermore the timing of the effects may be affected by individual differences in absorption of actives. The design of the study dictated that the participants ate a small meal following treatment (testing sessions took place between 12 and 4 pm). The meal was identical for each participant on each visit so will have minimally influenced between condition effects. However we cannot rule out the possibility that the meal will have slowed absorption such that the peak levels of rosmarinic acid will have been delayed and that the effects of the products would follow different patterns if taken with an empty stomach.

Turning to the effects of the lemon balm drink on anxiety, it is worth noting that the subjects were not selected on the basis of being anxious and/or stressed (any history of psychiatric disorders was an exclusion criterion). Furthermore the cohorts’ initial anxiety was within the normal range. Thus reduced anxiety associated with the treatments might reflect improvements in feelings described by the positive items on the state anxiety scale. These descriptors include “calm”, “secure”, “at ease”, “satisfied”, “comfortable”, “self-confident”, “relaxed”, “content’, “steady” and “pleasant”.

It is also possible, given the wide range of potentially active components, that the effects of lemon balm are mediated through a combination of mechanisms, with potential interactions with a number of neurotransmitter systems. Such a notion is wholly in keeping with that observed for other plant extracts [27]. Finally given the combination of lemon balm with fruit sweeteners in the treatments used here, we cannot rule out the possibility of synergistic interactions between these compounds.

Finally it is worth noting that the products were viewed as extremely acceptable to the participants. There were no adverse events during the trial and the participants remarked that they found the treatments appetising (although no systematic data were recorded); this is an important aspect of trials using real foods where compliance might otherwise be an issue.

## 5. Conclusions

Both active lemon balm treatments were generally associated with improvements in mood and/or cognitive performance, though there were some behavioral “costs” at other doses and these effects depended to some degree on the delivery matrix. The results indicate that Lemon balm delivered in foodstuffs can have positive behavioural effects which may be used in applied health settings.
